# Supplementary material for: AcuM and AcuK: The global regulators controlling multiple cellular metabolisms in a dimorphic fungus Talaromyces marneffei
Source: PLoS Negl Trop Dis. 2024 Sep 4;18(9):e0012145. doi: 10.1371/journal.pntd.0012145 (PMC11373862; doi:10.1371/journal.pntd.0012145)
Supplement: S3 Table — T. marneffei ATCC18224, Δ acuKΔ, and ΔacuM strains were cultivated under normal growth conditions (ANM broth) at 25°C for 36 hours. RNA samples were extracted, and qRT-PCR were conducted as described in materials and methods. (PDF) [file pntd.0012145.s003.pdf]

Table S3. Validation of transcriptomic data by qRT-PCR for selected genes. *T. marneffei* ATCC18224, Δ *acuKΔ*, and Δ*acuM* strains were cultivated under normal growth conditions (ANM broth) at 25°C for 36 hours. RNA samples were extracted, and qRT-PCR were conducted as described in materials and methods.

Mutant	Gene	Fold-change WT/mut	
		**RNA-Seq**	**qRT-PCR**
Δ*acuK*	*cytC*	0.10	0.46
	alxA	0.39	1.19
	fetC	3.16	3.47
	sidA	0.98	2.43
	sidF	1771.70	2.55
Δ*acuM*	*cytC*	0.12	0.60
	alxA	0.45	1.10
	fetC	3.72	0.73
	sidA	1.01	0.98
	sidF	1303.34	0.40
