# Supplementary figures and images for: AcuM and AcuK: The global regulators controlling multiple cellular metabolisms in a dimorphic fungus Talaromyces marneffei
Source: PLoS Negl Trop Dis. 2024 Sep 4;18(9):e0012145. doi: 10.1371/journal.pntd.0012145 (PMC11373862; doi:10.1371/journal.pntd.0012145)

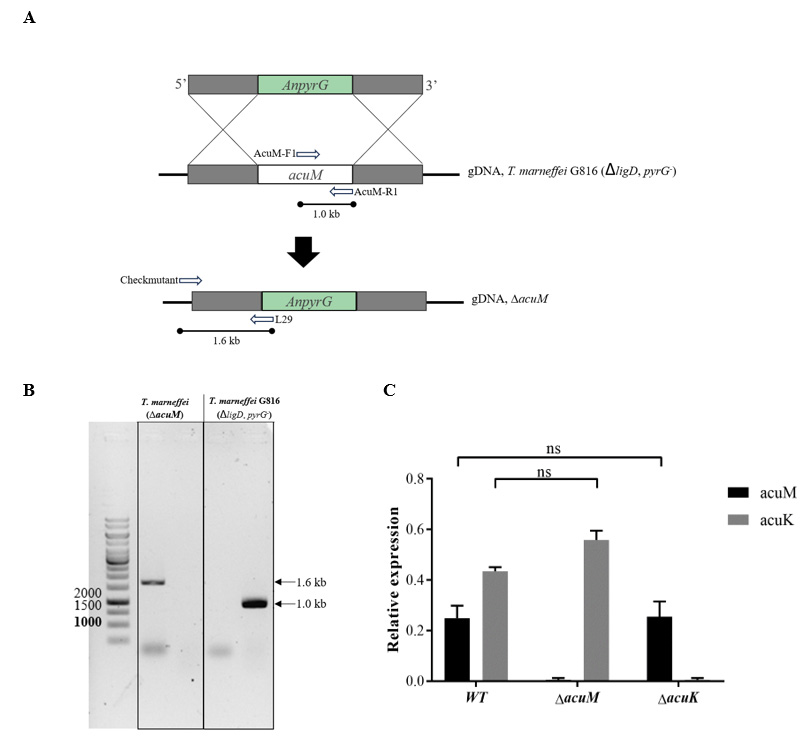

Supplement: S1 Fig — The acuM gene was deleted using the homologous recombination method. (A) The acuM knockout fragment was generated to delete the acuM gene from T. marneffei G816 genome (ΔligD, pyrG-) using the homologous recombination method. Primers used for diagnostic PCR were shown in blue arrows. Genomic DNA was extracted from transformants, and diagnostic PCR was performed using two primer pairs (Check mutant and L29; acuM-F1 and acuM-R1). (B) The 1.6-kb product was amplified from the first primer pair (Check mutant and L29), demonstrating the presence of AnpyrG marker at the acuM homologous integration locus. The 1.0-kb product was amplified from the second primer pair (acuM-F1 and acuM-R1), showing the presence of acuM gene. (C) qRT-PCR was performed to confirm the absence of acuK and acuM transcripts. (TIF) [file pntd.0012145.s007.tif]
